# TLR and NLRP3 inflammasome-dependent innate immune responses to tumor-derived autophagosomes (DRibbles)

**DOI:** 10.1038/cddis.2016.206

**Published:** 2016-08-04

**Authors:** Y Xing, R Cao, H-M Hu

**Affiliations:** 1 Minigene Pharmacy Laboratory, School of Life Science and Technology, China Pharmaceutical University, Nanjing, PR China; 2 Laboratory of Cancer Immunobiology, Robert W. Franz Cancer Research Center, Earle A. Chiles Research Institute, Providence Cancer Center, Portland, OR, USA

## Abstract

Autophagosomes derived from tumor cells, also referred to as defective ribosomal products in blebs (DRibbles), have been previously shown to stimulate potent T-cell responses and mediate tumor regression when used as therapeutic cancer vaccines in multiple preclinical cancer models. In this report, we investigated the underlining mechanisms by which DRibbles induced T-cell activation, particularly how DRibbles activated antigen-presenting cells (APCs). We found that DRibbles could induce a rapid differentiation of monocytes and DC precursor (pre-DC) cells into functional APCs. DRibbles triggered innate receptor signaling via Toll-like Receptors (TLR)-2, TLR4, TLR7, TLR8, and nucleotide-binding oligomerization domain-containing protein 2 (NOD2), but not TLR3, TLR5, or TLR9. DRibbles induced PBMCs to produce pro-inflammatory cytokines, such as IL-6, IL-10, TNF-*α*, and IL-1*β*. DRibbles induced IL-1*β* release from PBMC or THP-1 cells without LPS priming, but required the core machinery of NLRP3 inflammasomes. Active endocytosis was required for inflammasome activation and cross presentation, and blocking endosome acidification or the ER-associated degradation (ERAD) pathway resulted in opposite effects on these two processes. Our data show that DRibbles could induce strong innate immune responses via multiple pattern recognition receptors, and explain why DRibbles could function as excellent antigen carriers to induce adaptive immune responses to both tumor cells and viruses. In contrast to the well-established inhibitory effect of autophagy on the inflammasome activation of APCs, our study demonstrates that isolated autophagosomes (DRibbles) from antigen donor cells activate inflammasomes by providing first and second signals required for IL-1*β* production by PMBC.

Autophagy is a well-conserved cellular stress response pathway that enables organisms either as a unicellular eukaryote or complex metazoan to survive extreme conditions.^1^ For unicellular organisms, the survival is largely dependent on the autophagy pathway as a cell-intrinsic defense mechanism to acquire nutrients by recycling damaged proteins and other biomolecules. For multicellular organisms, autophagy serves an important role as a cell-intrinsic defense mechanism and a key regulator of inflammatory responses. The role of autophagy in the innate and adaptive immune responses is far more complex and less understood.

Originally regarded as a ‘bulk eater' system, for non-selective degradation and recycling pathway of expired or damaged cellular constituents, recent work demonstrates that autophagy is more of a ‘picky eater' involving various intracellular receptors responsible for substrate recognition and recruitment of autophagic machinery.^2^ Autophagy participates in the regulation of both apoptosis and inflammation (ref). The early work shows autophagy limits the activation and release of IL-1*β*.^3^

The innate immune system protects the host to limit cellular damage due to infection or other endogenous cellular stressors. Danger signals generated by cellular damage are recognized by host innate immune cells via innate immune signaling receptors, called pattern recognition receptors (PRRs). The inflammasomes are a group of multimeric protein complexes with the adaptor protein apoptosis-associated speck like CARD-domain-containing protein (ASC) and caspase-1 as the key components of the core inflammasome machinery.^4^ The danger signals stimulate the generation of inflammasome complexes in two steps. First, the danger factor binding to PRRs activates the transcription factor nuclear factor-*κ*B (NF-*κ*B), which induces the generation of a subunit of inflammasomes, such as NLRP3, and IL-1*β* precursor protein (pro-IL-1*β*) (Signal 1). Second, the ATP-dependent assembly of inflammasome triggers the process and release of the functional IL-1*β* via unconventional secretory pathway (Signal 2).^5^

Our previous study showed that autophagosomes act as antigen carriers that can be used as effective cancer vaccines.^6, 7^ However, the mechanism by which autophagosomes can help antigen-presenting cells (APCs) cross-present antigens to CD8^+^ T cells is not well understood, in mouse models or in humans. Tumor-derived autophagosomes (DRibbles) contain abundant materials like DNA, RNA, proteins, which could function as potent danger signals.^8^ In this study, we investigated whether DRibbles could deliver both signal 1 and 2 for NLRP3-dependent inflammasome activation. The role of endocytosis, protein retro-translocation and ERAD pathway in inflammasome activation was also examined.

## Results

### DRibbles induce efficient activation of human memory and effector T cells specific for CMV-pp65 antigen

**Figure 1:**
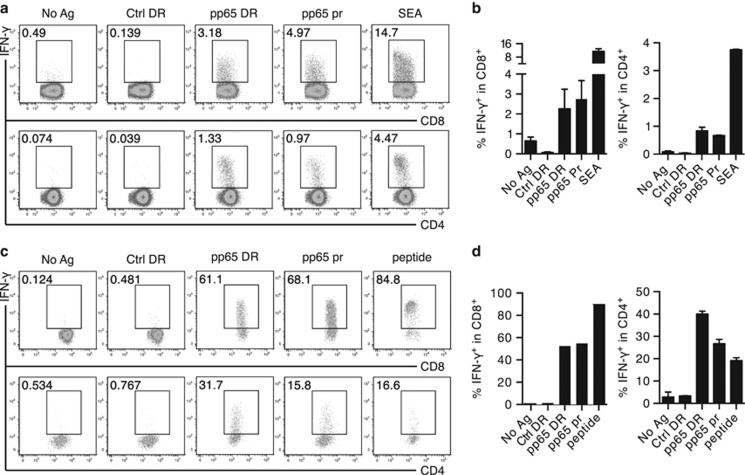
DRibbles induce efficient activation of human memory and effector T cells specific for CMV-pp65 antigen. (**a**) Dot plot and (**b**) bar graph of the frequency of IFN-*γ*^+^CD8^+^ T cells and IFN-*γ*^+^ CD4^+^ T cells in fresh PBMC, detected by ICS, after stimulation with either PBS, pp65 protein (10 *μ*g/ml), pp65 DRibbles (30 *μ*g/ml), control DRibbles (30 *μ*g/ml) or staphylococcal enterotoxin A (SEA) (10 *μ*g/ml). (**c**) Dot plot and (**d**) bar graph of the frequency of IFN-*γ*^+^CD8^+^ T cells and IFN-*γ*^+^CD4^+^ T cells in expanded pp65-specific T cells, detected by ICS, after re-stimulation by DCs loaded with either PBS, pp65 protein (10 *μ*g/ml), pp65 DRibbles (30 *μ*g/ml), control DRibbles (30 *μ*g/ml) or pp65 peptide (10 *μ*g/ml). Results are representative of two or more independent experiments

We previously demonstrated that direct loading of PBMCs with DRibbles derived from tumor cells expressing the CMV-pp65 antigen induced a potent antigen-specific T-cell recall response.^9^ Here, we obtained fresh PBMCs from different donors and repeated this experiment, using IFN-*γ* production as a read out of T-cell activation. Comparable levels of IFN-*γ*-producing CD4^+^ and CD8^+^ T cells were detected when PMBCs were directly stimulated with either pp65 DRibbles or recombinant pp65 protein (Figures 1a and b).

We next generated a short-term pp65-specific memory T-cell line by stimulating PBMCs *in vitro* with a peptide mix derived from pp65 sequences, followed by expansion with recombinant human IL-2 for 9–14 days (Supplementary Figure 1).^10^ First, we loaded monocyte-derived DCs with pp65 DRibbles, then used the loaded DCs to re-stimulate the pp65-specific memory T cells obtained by *in vitro* expansion.^11^ Using intracellular cytokine staining and flow cytometry analysis, we measured the number of antigen-specific IFN-*γ* producing T cells. We found that pp65 DRibbles were indeed cross-presented by DCs to the memory T cells, resulting in a substantial increase in IFN-*γ* production (40–80% in CD8^+^ T cells and 20–40% in CD4^+^ T cells; Figures 1c and d). In CD8^+^ T cells, DRibbles containing pp65 antigen-induced response levels similar to that induced by control pp65 protein. In CD4^+^ T cells, however, pp65 DRibbles induced a significantly stronger response, compared to the positive control pp65 protein.

Our data show that antigen-specific IFN-*γ* responses were obtained from both freshly isolated PBMCs and from *in vitro* expanded memory T cells. However, the magnitude of responses was markedly higher for the memory T cells (40–80% of IFN-*γ* producing CD8^+^ T cells), compared to effector T cells from PBMCs (around 0.5-5%). Given these robust and sensitive assays, we set forth to further explore how DRibbles deliver antigens, and how DCs capture DRibbles and process antigens for cross-presentation and robust T-cell activation.

### DRibbles induce production of pro-inflammatory cytokines from PBMCs

**Figure 2:**
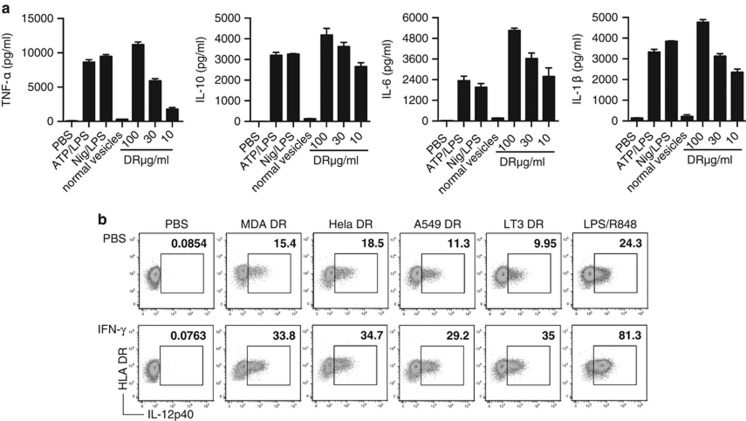
DRibbles induce pro-inflammatory cytokine production in human PBMCs. (**a**) PBMCs were incubated with 5 mM ATP, 5 *μ*M Nigericin, 100 *μ*g/ml, 30 *μ*g/ml or 10 *μ*g/ml MDA-MB-231 DRibble, or 100 *μ*g/ml normal vesicles overnight. The supernatant levels of TNF-*α*, IL-10, IL-6 and IL-1*β* were tested by ELISA. (**b**) Dot plot of IL-12p40 production by unprimed DCs or DCs primed with IFN-*γ*, as measured by ICS after stimulation with indicated DRibbles (100 *μ*g/ml) or LPS/R848 (10 ng/ml)/(50 ng/ml) overnight. Results are shown as the mean±S.D. and are representative of two or more independent experiments. PBMCs were from at least three donors

Given our results that DRibbles with pp65 antigen can generate potent T-cell responses, we further investigated whether DRibbles could induce innate immune responses from PBMCs. We incubated PBMCs overnight with DRibbles at various concentrations and collected the cell culture supernatant for ELISA analysis of typical inflammatory cytokines. As a negative control, we used normal vesicles, which were collected in a similar manner to the DRibble vaccine but without pharmacological treatments that block the proteasome. There was a dose-dependent release of inflammatory cytokines TNF-*α*, IL-6, IL-10, and IL-1*β* after stimulation with DRibbles (Figure 2a). When PMBC were stimulated with 100** ***μ*g/ml DRibbles, the levels of TNF-*α*, IL-6, IL-10 and IL-1*β* were higher than that induced by the positive controls of ATP/LPS or Nigericin/LPS; normal vesicles induced very low levels of these cytokines.

Next, we tested the effect of priming DCs with IFN-*γ* before stimulation with DRibbles. When DCs were stimulated with DRibbles alone, isolated from a number of different sources, ~10–20% produced IL-12p40, based on an intracellular staining assay. This percentage increased to 30–35% when DCs were primed with IFN-*γ* before stimulation with DRibbles (Figure 2b). These results show that DRibbles are potent activators of DCs and inducers of both pro-inflammatory cytokines (IL-1*β*, 6, 12, and TNF-*α*) and anti-inflammatory cytokine (IL-10).

### DRibbles trigger multiple Toll-like receptor (TLR) signals

**Figure 3:**
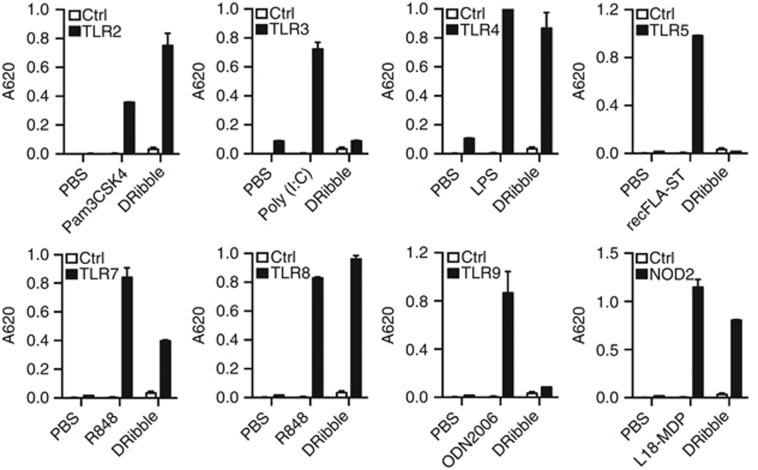
DRibbles trigger multiple Toll-like receptor signals. Human TLR2, TLR3, TLR4, TLR5, TLR7, TLR8, TLR9 and NOD2 modified HEK-Blue cells were incubated for 12 h with PBS, 100 *μ*g/ml UbiLT3 DRibbles, or with their respective agonists: 10 ng/ml Pam3CSK4, 1 *μ*g/ml Poly (I:C), 100 ng/ml LPS, 10 ng/ml recFLA-ST, 50 ng/ml R848, 1 *μ*g/ml R848, 0.3 *μ*g/ml ODN2006, or 100 ng/ml L18-MDP as positive control. The level of SEAP in the supernatant following TLR/NOD activation was tested by reading the optical density (OD) at 620 nm, according to the QUANTI-Blue detection assay. Results are shown as the means±S.D. and are representative of two or more independent experiments

As DRibbles were able to efficiently stimulate pro-inflammatory and anti-inflammatory cytokines, we tested whether DRibbles could act through TLRs. To determine whether and which TLRs were involved in the release of DRibble-induced inflammatory cytokines, we exploited TLR reporter cell lines that express individual TLRs. We incubated HEK-Blue cells expressing human TLR2, TLR3, TLR4, TLR5, TLR7, TLR8, TLR9, or the intracellular PRR protein NOD2 with 100 *μ*g/ml DRibbles derived from the mycoplasma free lung cancer cell line UbiLT3. HEK-Blue cells are engineered to stably co-express a human TLR/NOD and NF-*κ*B-inducible secreted embryonic alkaline phosphatase (SEAP) reporter gene. The results showed that DRibbles were potent agonists for TLR2, TLR4, TLR7, TLR8, and NOD2, but not for TLR3, TLR5, and TLR9 (Figure 3). A similar pattern was observed when DRibbles from different cell lines were used (not shown). These results show that DRibbles are capable of acting through multiple TLRs, as well as the PRR NOD2, which likely contributes to release of inflammatory cytokines.

### DRibbles activate NLRP3-dependent inflammasomes

**Figure 4:**
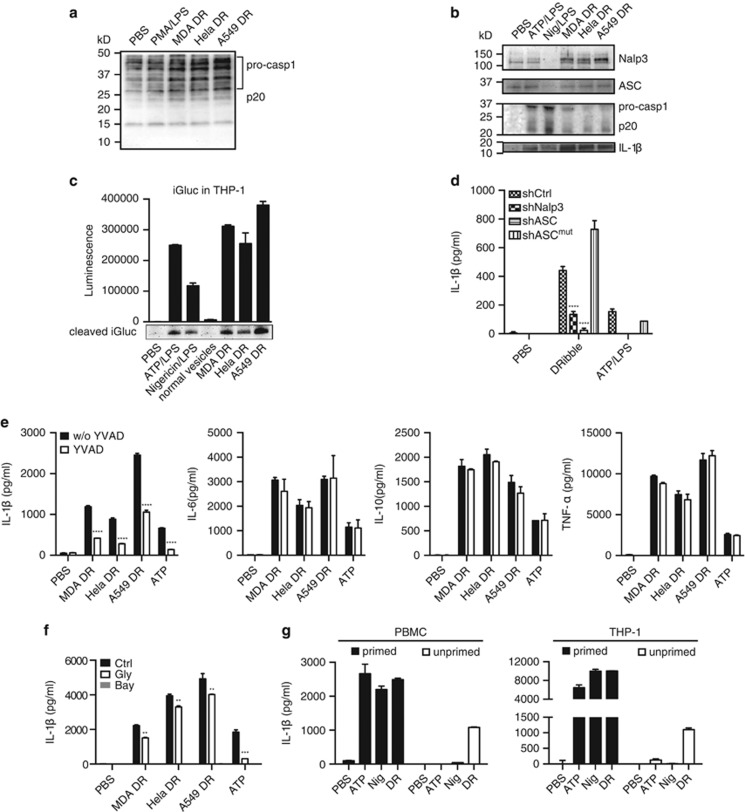
DRibbles activate NLRP3-dependent inflammasome. (**a**) THP-1 cells were treated with PMA/LPS (100 nM)/(10 ng/ml) or indicated DRibbles for 6 h, caspase-1 in cell lysate was measured by western blot. (**b**) PBMC were incubated with ATP/LPS (5 mM)/(10 ng/ml), Nigericin/LPS (5 *μ*M)/(10 ng/ml) and DRibbles (100 *μ*g/ml) made from MDA-MB-231, Hela and A549 cells overnight. Caspase-1 and IL-1*β* in supernatant, and Nalp3 and ASC in cell lysate were detected by western blot. (**c**) Primed iGluc reporter gene modified THP-1 cells were incubated with ATP/LPS (5 mM)/(10 ng/ml), Nigericin/LPS (5 *μ*M)/(10 ng/ml), DRibbles (100 *μ*g/ml) made from MDA-MB-231, Hela and A549 cells and normal vesicles (100 *μ*g/ml) from MDA-MB-231 cells overnight, the luciferase activity was measured and the cleaved iGluc in supernatant was detected by western blot with anti-Flag antibody. (**d**) Secreted IL-1*β* levels were evaluated in PMA/LPS primed THP-1 cells stably expressing control shRNA (shCtrl), shNLRP3, shASC or the re-expression of a knockdown resistant ASC cDNA (shASC^mut^). (**e**) Primed THP-1 treated with and without caspase-1 inhibitor YVAD at 50 *μ*M for 30 min, then stimulated with 5 mM ATP and 100 *μ*g/ml DRibbles overnight. (**f**) Primed THP-1 treated with and without NLRP3 inflammasome inhibitor 25 *μ*g/ml glybenclamide and 1 *μ*M Bay11-7082 for 30 min, then stimulated with 5 mM ATP and 100 *μ*g/ml DRibbles overnight. (**g**) PMA/LPS primed/unprimed THP-1 cells were stimulated with indicated stimulus at described concentration. The supernatant IL-1*β*, IL-6, TNF-*α* and IL-10 was detected by ELISA. Results are shown as the means+S.D. and representative of two or more independent experiments. PBMCs are from ⩾3 donors

Consistent with the DRibbles' ability to activate multiple TLRs, caspase-1 protein was greatly induced by incubating unprimed THP-1 cells with DRibbles compare to phorbol myristate acetate (PMA)/LPS (Figure 4a). DRibbles also induced synthesis of NLRP3 and ASC protein and activation of pro-caspase-1 and IL-1*β* from the PBMC (Figure 4b). We also examined the IL-1*β* induced by DRibbles with a pro-IL-1*β*-Gaussia luciferase (iGluc) gene transfected reporter THP-1 cell line in which pro-IL-1*β*-dependent formation of protein aggregates renders Gluc enzyme inactive, the cleavage of IL-1*β* by caspase-1 leads the biosensor protein to be monomerization, then the fusion protein gains a strong luciferase activity.^12^ By detecting the luminescence intensity in the co-culture supernatant and also the cleavage iGluc with western blot, the results showed DRibble-induced strong luminescence signal, which was comparable to ATP/LPS and Nigericin/LPS, but the control normal vesicles hardly activated these responses (Figure 4c).

Because DRibbles contain many endogenous danger signals, we suspect that NLRP3 inflammasome is involved in the IL-1*β* secretion by THP-1 cells induced by DRibbles. To examine this possibility, we used the THP-1 cells in which NLRP3 (shNLRP3) or ASC (shASC) expression were specifically knocked down by siRNAs. The ability of DRibbles to stimulate IL-1*β* activation was found greatly reduced from cell lines with NLRP3 or ACS knockdown (Figure 4d). Reassuringly, the responses to DRibbles or ATP/LPS were fully restored in the ASC knockdown and re-expressing cells (shASCmut). To confirm that IL-1*β* secretion is mediated by caspase-1, we added caspase-1 inhibitor, YVAD, during the incubation of THP-1 with DRibbles.^13, 14^ The results showed that IL-1*β* production was specifically inhibited by YVAD, however, the TNF-*α*, IL-6 and IL-10 response was not affected (Figure 4e). NLRP3 inflammasome inhibitor Glybenclamide and Bay11-7082 also blocked the DRibbles IL-1*β* (Figure 4f).^15, 16^

Unlike ATP or Nigericin, DRibbles could stimulate PMA/LPS primed and unprimed THP-1 to produce IL-1*β*, and as the same in PBMC (Figure 4g). Therefore, combined with the multiple TLR agonist effect and the capacity to induce NLRP3 proteins, DRibbles are capable to provide both signal 1 and 2 for the assembly and stimulation of NLRP3-ASC-Caspase-1 inflammasome.

### Autophagosomes not exosomes are the major and potent inducers of IL-1*β* in DRibbles

**Figure 5:**
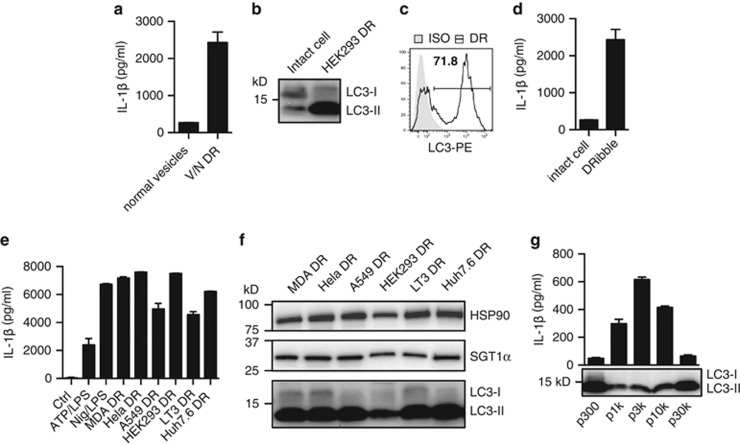
Autophagosomes not exosomes are the major and potent inducers of IL-1*β* in DRibbles. (**a**) A total of 100 *μ*g/ml normal vesicles with no drug treated and bortezomib/NH_4_Cl-treated DRibbles from UbiLT3 cells incubated with primed THP-1 overnight. The supernatant IL-1*β* was tested by ELISA. (**b**) LC3 status of HEK293 DRibbles and intact cells was measured by western blot and (**c**) DRibbles labeled with LC3-PE or isotype control was detected by flow cytometry. (**d**) Intact cells (100 *μ*g/ml) and HEK293 DRibbles (100 *μ*g/ml) incubated with primed THP-1 overnight. The supernatant IL-1*β* was tested by ELISA. (**e**) LC3, HSP90 and SGT1 in indicated DRibbles were measured by western blot. (**f**) Primed THP-1 was stimulated with 5 mM ATP/LPS, 5 *μ*M Nigericin/LPS and 100 *μ*g/ml DRibbles made from indicated cells overnight, the supernatant IL-1*β* was tested by ELISA. (**g**) Primed THP-1 was stimulated with UbiLT3 pellet fractions (p300, p1k, p3k, p10k, p30k) at 100 *μ*g/ml overnight, the supernatant IL-1*β* was tested by ELISA. LC3 status of different cell fractions was measured by western blot. Results are shown as the means+S.D. and representative of two or more independent experiments

To avoid the fake inflammatory response from endotoxins, which may involve during the DRibbles preparation process, all the cell lines were cultured in endotoxin free system and detected to be mycoplasma free, and maintained in anti-mycoplasma reagent added medium. We also generated normal vesicles as control, which were prepared the same way as DRibbles we used as vaccine from UbiLT3 cell lines but without bortezomib and NH_4_Cl treated. After inflammasome detection assay, we found only DRibbles could induce IL-1*β* secretion (Figure 5a).

As we know, IL-1*β* is the major inflammatory cytokine that is produced and released mainly from myeloid cells sense microbial infections or endogenous danger molecules. Necrotic cells release multiple alarmins as endogenous danger molecules and are reported to cause inflammatory responses and IL-1*β* releasing. The relationship between vesicles released from necrotic cells and DRibbles are not known. Thus, it is important to investigate whether DRibbles-mediated IL-1*β* response is caused by damaged cells.

DRibbles contain mis-folded proteins and damaged organelles.^17^ DRibbles are secreted vesicles with enriched autophagosomes as indicated by the greater amount of LC3-II found in DRibble preparation (Figures 5b and c). First, we compared the production of IL-1*β* induced by DRibbles with that induced by whole cells. The data showed isolated DRibbles could induce much higher IL-1*β* production as compared to whole cells, when the whole-cell equivalent amount of DRibbles were used to stimulate THP-1 (Figure 5d). Then, we compared the ability of DRibbles made from different tumor cell lines to stimulate the IL-1*β* production from THP-1. We found that all DRibbles from six cell lines could efficiently activate IL-1*β* release (Figure 5e), western blot analysis of the six kinds of DRibbles showed similar patterns of LC3 status and they all contained HSP90 and SGT1, which are important sensor protein for inflammasome activation (Figure 5f). These cell lines include breast, lung, liver, and kidney cell lines.

To further fractionate the DRibble preps according to their sizes, a modified differential centrifugation method was developed. In addition to the original low speed centrifugation step (300 × *g*) for the clearance of large cellular debris and high speed step (10 000 ×  *g*) to pellet DRibbles, two more 1,000 × *g* and 3 000 × *g* steps were added to further fractionate DRibble preps, and an extra ultra-high speed (30 000 × *g*) centrifugation step to pellet the exosomes from the DRibble-free supernatant. Western blot analysis with anti-LC3 antibodies revealed that a dominant LC3-II protein was detected in all fractions, however, the IL-1*β* inducing activity was limited to the pellets produced by 1 000 × *g*, 3 000 × *g*, and 10 000 × *g* centrifugation (Figure 5g). The fractions that contained whole cells and large cellular debris (300 × *g* pellet) or the DRibble-free exosome pellet (30 000 ×  g pellet) induced a minimal IL-1*β* release. On the basis of these observations, we concluded that autophagosomes released from many different tumor cells were potent inducers of IL-1*β*.

### Protein fraction in DRibbles is important for IL-1*β* induction

**Figure 6:**
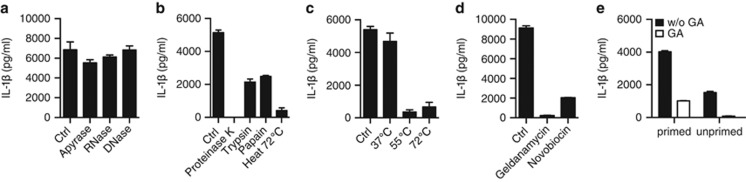
Protein fraction in DRibbles is important for IL-1*β* induction. (**a**) Apyrase, RNase and DNase; (**b**) Proteinase K, trypsin, papain and 72 °C heat; (**c**) 37, 55, 72 °C water bath; (**d**) HSP90 inhibitor geldanamycin (GA) and Novobiocin treated for 30 min before DRibbles were incubated with primed THP-1 overnight. (**e**) Primed/unprimed THP-1 were stimulated with GA-treated/untreated 100 *μ*g/ml DRibbles overnight. The supernatant IL-1*β* was tested by ELISA. Results are shown as the means+S.D. and representative of two or more independent experiments

Autophagosome contains DNA, RNA, mis-folding protein, ATP and various cell components. Some of these fractions are known to cause inflammasome response themselves. Next, we detected which components in DRibbles mainly effected on inducing innate immune responses. ATP, DNA and RNA removed DRibbles, which were respectively pre-treated with apyrase, DNase, RNase, could still induce IL-1*β* response as control DRibbles with no significant difference (Figure 6a). But when we digested the protein component with different proteolytic enzyme or by heating at different temperature, DRibbles more or less lost their efficiency to arouse IL-1*β* activation (Figures 6b and c). So we concluded that protein mainly caused the inflammasome induction in DRibbles.

The molecular chaperone HSP90 (heat shock protein 90 kD) stabilizes NLR protein in macrophage and also ubiquitous molecules in DRibbles. HSP90 in DRibbles is necessary for the vaccine T-cell response in mouse in our early study, and we also found HSP90 dysfunction in DRibbles would inhibit human T-cell IFN-*γ* response *in vitro* (Supplementary Figures 2a and b).^18^ So we considered HSP90 in DRibbles might also help delivering NLR ligands in ubiquitinated proteins enriched DRibbles to respond immune cells. DRibbles were incubated for 30 min with HSP90 inhibitors geldanamycin (GA), which binds to N-terminal ADP/ATP-binding pocket of the HSP90;^19^ and novobiocin, which interacts with C-terminal ATP-binding domain of HSP90.^20^ IL-1*β* secretion markedly declined in HSP90 inhibitor-treated DRibbles than in untreated control group (Figure 6d). For further detection, we found IL-1*β* production was significantly reduced both in PMA/LPS primed and unprimed THP-1 (Figure 6e). From these data, we concluded that HSP90 was required for both signals of the DRibbles-inflammasome activation and we hypothesis that some HSP90 assembled ubiquitinated proteins in DRibbles might be the direct adaptor of NLR.

### DRibbles access cytosol via different routes for the inflammasome activation and proteasome-mediated degradation after phagocytosis of DRibbles

**Figure 7:**
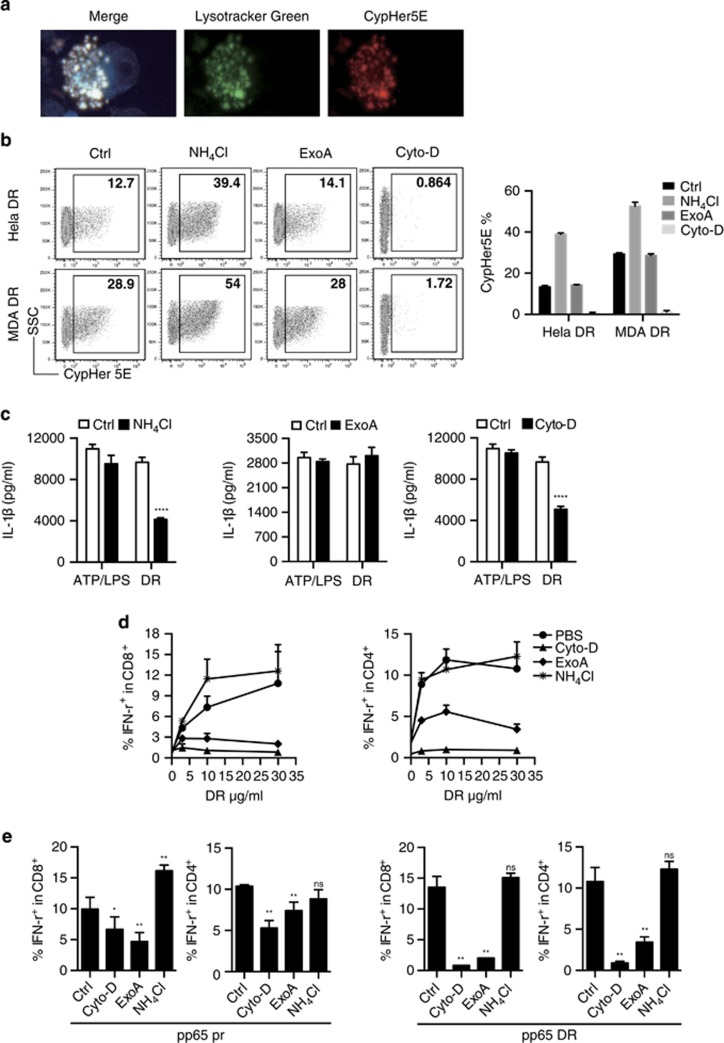
Endocytosis was necessary in the process of DRibbles-inflammasome response. (**a**) CypHer5E-labeled DRibbles and lysotraker Green-labeled lysosome were collocated in THP-1 cells after 6 h incubation, and the nucleus were labeled with DAPI. (**b**) The endocytosis frequency of DRibbles by THP-1 cells was measured by FACS. DCs were pre-treated with ExoA (1 *μ*M), Cytochalasin-D (Cyto-D, 1 *μ*g/ml), and NH_4_Cl (20 *μ*M) for 30 min, then incubated with CypHer5E stained DRibbles (100 *μ*g/ml) overnight. (**c**) Primed THP-1 cells were pre-treated with cytochalasin-D, ExoA, and NH_4_Cl for 1 h, then stimulated with ATP/LPS (5 mM)/(10 ng/ml) and DRibbles (100 *μ*g/ml) overnight. The supernatant IL-1*β* was detected by ELISA. (**d**) The frequency of IFN-*γ*^+^CD8^+^, IFN-*γ*^+^CD4^+^ T cells induced by pp65 DRibbles at indicated doses were measured by ICS, and (**e**) pp65 protein (10 *μ*g/ml) and pp65 DRibbles (10 *μ*g/ml). Results are shown as the means+S.D. and representative of two or more independent experiments. PBMC is from ⩾3 donors

Inflammasome activation could be induced by direct binding of particles to cell surface, by phagocytosis of DRibbles, or by membrane fusion between DRibbles and monocytes. Both phagocytosis and membrane fusion, but not direct surface binding, require active actin polymerization. To visualize the phagocytosis of DRibbles, we labeled DRibbles with pH-sensitive cyanine dye CypHer5E, which is minimally fluorescent at neutral pH, but highly fluorescent in the acidic environment of phagosomes.^21^ The fluorescent image showed CypHer5E-labeled MDA DRibbles were located in the lysosome in THP-1 cells after 6 h incubation (Figure 7a). By flow cytometry analysis, phagocytosis of Hela and MDA DRibbles by THP-1 cells was completely diminished by treatment with the actin polymerization inhibitor cytochalasin-D (Figure 7b). IL-1 *β* release from THP-1 was significantly, but only partially reduced by cytochalasin-D treatment of THP-1 (Figure 7c). Because NLRP3 inflammasomes reside exclusively in cytosol, we investigate the possibility that the content of DRibbles, particularly the endoplasmic-reticulum-associated protein degradation (ERAD) pathway related substrates in the DRibbles, are released into cytosol and activate inflammasome via the retro-translocation pathway of ERAD. Previously, we have shown that cross-presentation of antigens in DRibbles was effectively suppressed using the known inhibitor of this pathway, ExoA, a *Pseudomonas aeruginosa*-derived toxin.^18^ Again, we showed that treatment of PBMC with ExoA, which specifically blocks the endosome to cytosol protein translocation (Figure 7b) and cross-presentation (Figures 7d and e); blocking ERAD with ExoA had no effect on the IL-1*β* release from PBMC (Figure 7c). Thus, the ERAD proteasome substrates in the DRibbles provide the antigens, but they are not activators of inflammasome.

To determine the involvement of lysosomes in the DRibble-induced IL-1*β* release, we used NH_4_Cl to block the acidification of phago-lysosomes. NH_4_Cl treatment of PBMC effectively but partially blocked the IL-1*β* release (Figures 7b and c), it is likely that lysosomes are involved in the release content of DRibbles that stimulate NLRP3 inflammasomes. The concentration of three compounds used in above experiments did not affect the IL-1*β* release from PBMC that were treated in parallel with ATP/LPS, indicating lysosomes were involved in the upstream events of IL-1*β* processing induced by DRibbles rather on the release of processed IL-1*β*. Reduced inflammasome activation by NH_4_Cl treatment seems to benefit cross-presentation and CD8^+^ T-cell activation, but it has no effect on CD4^+^ T-cell activation (Figures 7d and e).

## Discussion

Autophagy is an extremely important pathway that regulates cell-intrinsic defense, inflammation, innate and adaptive arms of immunity.^22^ Depending on the experimental systems, previous studies show autophagy assists both pro-inflammatory and anti-inflammatory processes. Autophagy delivers PAMPs from cytosol to endosome TLRs, and autophagy contributes to the unconventional secretion of IL-1*β* and also IL-18. Autophagy suppresses inflammasome activation by fusing with lysosomes, then clearing sources of endogenous NLRP3 inflammasome agonists and digesting some of the inflammasome complex components.^23, 24^ Dysfunction of autophagy increases inflammasome related IL-1*β* production.^3, 25^

Autophagy in immune cells affects their development and function.^26^ Autophagosome from damaged cell could act as immune response inducer also.^27^ Earlier, we demonstrate that drug derived autophagosome-DRibbles induced activation and maturation of DCs, antigen cross-presentation, and T-cell activation of CD4^+^ and CD8^+^ memory T cells.^9^ Here, we demonstrated that autophagy was critically involved in the transfer of antigens from antigen donor cells into DCs and secretory autophagosomes can serve as efficient antigen carriers and vaccines for immunotherapy of cancers. With all of these effects of DRibbles on T-cell activation, we discovered this autophagosome structure-based vaccine had the ability to elicit multiple TLRs signals as we described, and induced pro-inflammatory cytokines production including NLRP3 inflammasome-dependent IL-1*β*.

MHC-restricted antigen presentation needs cellular translocation events to generate antigenic fragments, and then the products are delivered to the APC surface for T-cell recognition. MHC class I and II molecules present antigenic proteolysis products of proteasome and lysosome to CD8^+^ cytotoxic and CD4^+^ helper T-cells, respectively.^28^ For non-professional APCs, such as carcinoma cells, MHC-I present antigenic derived from proteins synthesized by endogenous ribosome – a process generally referred as direct presentation. On the other hand, professional APCs, such as DCs, can also endocytose exogenous antigens synthesized by ribosomes of antigen donor cells and present antigens via the MHC-II pathway. Because naive T cells are typically circulating in lymphatic tissues, professional APCs are responsible for antigen up-taking and the priming of T-cell responses against pathways do not replicate in them. For MHC-I restricted presentation of exogenous antigens, a process referred as cross-presentation, happens only efficiently enough in a specialized subsets of DCs.^29^ Thus, these subsets of DCs have a critical role in the generation of CTL responses and immune surveillance against intracellular infections and tumor cells. The cross-presentation typically requires functional proteasomes of APC but not donor cells. Antigen donor cell derived proteasome substrates, but not products, are efficiently cross-presented and the efficiency of antigen cross-presentation depends on the protein levels-proteasomal inhibition enriched ubiquitinated proteins, including defective ribosomal products.^30^

The autophagosome membrane transferred out layer of the cup into inner layer and released as defective ribosomal products-containing autophagosome-rich blebs (DRibbles). DRibbles can be endocytosed in a CLEC9A-dependent fusion by APCs. Our research revealed DRibbles induce intensive T-cell response especially when they were presented by CD141^hi^CLEC9A^+^ professional cross presentation DC in human and CLEC9A^+^ DC in mouse (manuscript in preparation). The chemokine receptor XCR1, in addition to CLEC9A, is expressed by both human and mouse cross-presenting DC subset.^31^ We suggest to use xDC as the unique nomenclature to distinguish this subset phenotypically and functionally for conventional DCs and plasmacytoid DCs.

This subset of DCs is equipped with specialized antigen process and presentation machinery that enable them to cross-present exogenous antigens more efficiently than other subsets of DCs or macrophages. The recruitment of NADPH oxidase 2 (NOX2) to the phagosome membrane resulted in production of ROS and slow acidification of phagosomes.^32^ In addition, Serbian identified a Sec22b-dependent recruitment of ER-resident proteins to phagosome via the ER–Golgi intermediate compartment (ERGIC) as the critical step for cross presentation. TLR signaling is known to be import for cross-priming of naive CD8^+^ T cells; however, how TLR signaling influences the generation of MHC-I peptide complexes is far from clear. Naif-Gupta recently demonstrated a TLR-MyD88-IKK2 axis dependent MHC-I trafficking from endosomal recycling compartment (ERC) as another important step of cross presentation.^33, 34^ This TLR-dependent MHC-I trafficking from ERC (TRIF-independent) was distinct from TLR-independent Sec22b-mediated recruitment of MHC-I loading complex from ERGIC. Most recently, Zehner showed that TLR-TRIF axis mediated Sec61 translocation from ER to endosomes is required for antigen translocation and cross presentation.^35, 36^

Similar to what described for particulate or cell-associated antigens, cross-presentation of DRibbles depended on endocytosis, Sec61/p97-dependent retro-translocation, and ERAD pathways. Our result further expanded our previously published observations using mouse bone marrow derived DCs.

Consistent with the high level expression of TLR3 by xDC, addition of TLR3 agonist to DRibbles markedly augmented the T-cell activation. One possible consequence of TLR3 signaling is the increased antigen delivery from endosome into cytosol via the Sec61 channel. Indeed, cross-presentation of DRibbles was complete abolished when Sec61 channel was blocked with ExoA, a known inhibitor of Sec61 and antigen cross presentation.^35^ DRibbles also contain endogenous TLR agonists, which efficiently stimulate TLR2, TLR4 but not TLR3, thus it is not surprising that LPS or TLR2 agonist had no effect on DRibble-Induced T-cell activation. In addition, our results with poly(I:C) indicated that a direct co-stimulation of memory/effector CD8^+^ T cells could contribute significantly to IFN-*γ* production.

## Materials and Methods

### PBMCs, cell lines, cell culture

HEK 293T were cultured in DMEM (Lonza, Basel, Switzerland, 12-604 F) supplemented with 2 mM l-glutamine, 100 units/ml penicillin and 100 *μ*g/ml streptomycin (Invitrogen, Carlsbad, CA, USA, 10378), 1 mM sodium pyruvate (Lonza, 13-115E), and 10% fetal bovine serum (FBS, Invitrogen, 16000044). UbiLT3 (human lung cancer cell line), MDA-MB-231, Hela and A459 human cancer cell lines were cultured in RPMI 1640 complete medium (CM) (Lonza, 12-702Q) supplemented with 10% fetal bovine serum (FBS, Invitrogen, 16000044), 50 *μ*g/ml Gentamicin (Life Technologies, Carlsbad, CA, USA, 15750-078), 2 mM l-glutamine and 1 mM sodium pyruvate (Lonza, 13-115E). THP-1 lines were cultured in RPMI 1640 complete medium. ASC (shASC), NLRP3 (shNLRP3) knockdown and ASC re-expressing (shASC^mut^) THP-1 lines were generous gifts from Dr Jenny P-Y Ting. All cell lines were tested mycoplasma free. Human PBMCs and monocytes were separated from whole blood cells of health donors. Frozen PBMCs were thawed, rested and maintained in CM mixed with X-VIVO 15 (Lonza, 04-418Q) at the ratio 1:1 (CMX) for 24 h. All cell culture medium was added with 50 *μ*g/ml plasmocure (Invivogen, ant-pc) to prevent mycoplasma contaminant. Written informed consent was obtained from all donors in accordance with the Declaration of Helsinki. All studies were approved by the institutional review board (IRB) of Providence Portland Medical Center.

### Expansion of pp65 antigen-specific T cells

PBMCs were stimulated with MHC-I restricted CEF peptide pool (Panatecs, Heilbronn, Germany, PA-CEF-002) at 20 ng/ml work concentration of each peptide in CMX media for about 48 h. Primed T cells were expanded in CMX medium supplemented with 1000 U/ml human IL-2 (Peprotech, Rocky Hill, NJ, USA, 200-02) for further 10-12 days. Fresh medium with IL-2 was added to the culture system every 3 days when needed.

### Inflammasome stimulation

THP-1 cell lines were primed with 100 nM PMA (InvivoGen, San Diego, CA, USA, tlrl-pma) and 10 ng/ml LPS (InvivoGen, tlrl-eblps) overnight, and PBMCs were primed with 10 ng/ml LPS for 3 h, then stimulated with 5 mM ATP (Sigma-Aldrich, St. Louis, MO, USA), 5 *μ*M Nigericin (InvivoGen, tlrl-nig), or DRibbles at indicated dose overnight. Inflammasome inhibitors 50 *μ*M Ac-YVAD-cmk (Sigma-Aldrich, SML0429), glybenclamide (Sigma-Aldrich, G0639) and Bay11-7082 (Sigma-Aldrich, B5556) were added to THP-1 or PBMC separately 30 min before stimulation.

### Preparation of DRibbles

Indicated cells were treated with 200 nM bortezomib (Millennium Pharmaceuticals, Inc., Cambridge, MA, USA) and 20 mM ammonium chloride (Sigma-Aldrich, A9434) for 24 to 48 h, and DRibbles were prepared as described previously. Cells and debris were separated by centrifugation at 300 × *g* for 10 min. DRibbles were collected from suspension after centrifugation at 10 000 × *g* for 15 min. The total amount of protein in DRibbles was quantified with BCA protein assay kit according to the manufacturer's protocol (Thermo Scientific, Waltham, MA, USA, 23225). LC3-I and II was detected by western blot with LC3B antibody (Cell Signaling Technology, Danvers, MA, USA, 2775 S). Flow cytometric analysis of DRibbles using LC3B antibody compared to an isotype negative control antibody.

### Measurement of mature caspase-1 and inflammatory factors

Culture supernatants IL-1*β*, IL-6, IL-10, TNF-*α* levels were measured by IL-1*β* (R&D Systems, Minnneapolis, MN, USA, DY201), IL-6 (R&D Systems, DY206), IL-10 (R&D Systems, DY217B), TNF-*α* (R&D Systems, DY210) ELISA kit. Caspase-1, ASC, Nalp3 and IL-1*β* was detected by western blot with anti-caspase-1 (Cell Signaling Technology, 2225S), anti-ASC (Millipore, Billerica, MA, USA, AB3607), anti-Nalp3 (Cell Signaling Technology, 13158S), and anti-IL-1*β*.

### Detection of IL-1*β* signal by iGluc reporter gene transfected THP-1 line

THP-1 cells stable expressing iGluc with a Flag tag added to the C-terminal were generated and primed with PMA/LPS overnight, then stimulated with 5 mM ATP (Sigma-Aldrich), 5 *μ*M Nigericin (InvivoGen, tlrl-nig), or indicated DRibbles for 12 h, the supernatant luciferase activity was examined with a Gaussia Luciferase Glow Assay Kit (Thermo Scientific, 16161). The cleavage iGluc was detected by western blot with anti-Flag antibody. The iGluc DNA sequence was a generous gift from Dr Veit Hornung.

### Flow cytometry analysis and intracellular cytokine staining

After stimulation, PBMC or antigen-specific T cells were collected into FACS tubes and stained with live/dead fixable dead cell staining dye (Invitrogen, L34957) and stained with antibodies against CD3 (PerCP, Invitrogen, MHCD0331), CD4 (PE, Invitrogen, MHCD0404), CD8 (FITC, Invitrogen, MHCD0801), and IFN-*γ* (APC, Invitrogen, MHCIFG05). Intracellular IL-12p40 of DCs was measured by standard ICS staining process with the antibodies against HLA-DR (PerCP, Biolegend, San Diego, CA, USA, 307628) and IL-12p40 (PE, Biolegend, 505203). All FACS data were analyzed by a custom-build LSRII flow cytometer (BD Bioscience, Franklin Lakes, NJ, USA) or LSR Fortessa instrument (BD Biosciences). Data were collected with BD FACSDiva software (San Jose, CA, USA) and analyzed with Treestar Flowjo software (Flowjo, LLC, Ashland, OR, USA).

### Analysis of DRibbles phagocytosis utilizing a pH-sensitive fluorescent dye

DRibbles (1 mg/ml) were incubated with pH-sensitive cyanine dye CypHer5E (GE Healthcare, Chicago, IL, USA, PA15401) at 10 *μ*g/ml for 15 min on ice, then washed twice with PBS to remove the extra free dye. Stained DRibbles were added into 5 × 10^5^ THP-1 cells in 1 ml culture system in 24 well plate. After 12–24 h, detected the fluorescence intensity and frequency of THP-1 by flow cytometry. FACS data were analyzed by LSRII flow cytometer (BD Bioscience). Data were collected with BD FACSDiva software and analyzed with Treestar Flowjo software.

CypHer5E-labeled DRibbles (100 *μ*g/ml) were incubated with 5 × 10^5^ lysotracker green (Thermo Scientific, L7526) stained THP-1 cells in 500 *μ*l culture medium for 6 h, then cells were harvested and stained with DAPI (Thermo Scientific, R37606). DRibbles in cells were observed under fluorescence microscope, images were acquired on the Zeiss ZEN lite Auto imaging system using a 20 × objective.

### Statistical analysis

Unpaired *t*-test or one-way ANOVA was performed using Prism (GraphPad Software Inc., San Diego, CA, USA). *P<0.05; **P<0.01; ***P<0.001; ****P<0.0001; ns, not significant. *P*<0.05 was considered significant.

## Abbreviations

DC: dendritic cell
APC: antigen-presenting cell
ERAD: ER-associated degradation
NLR: NOD-like receptors
PRR: pattern recognition receptor
DAMPs: damage-associated molecular patterns
PAMPs: pathogen-associated molecular patterns
TLR: Toll-like receptors
(NOD2): Nucleotide-binding oligomerization domain-containing protein 2
ASC: apoptosis-associated speck like CARD-domain-containing protein
NLRP3: NACHT, LRR, and PYD domains-containing protein 3/cryoporin
IFN: interferon
IL: interleukin
CTL: cytotoxic T lymphocyte
PBMC: peripheral blood mononuclear cell
CEF: cytomegalovirus, Epstein–Barr virus, influenza virus
CMV: cytomegalovirus
ExoA: Exotoxin A
ERC: endosomal recycling compartment
ERGIC: ER–Golgi intermediate compartment
NOX2: NADPH oxidase 2
BCA: bicinchoninic acid
ICS: intracellular staining
