# Supplementary material for: TLR and NLRP3 inflammasome-dependent innate immune responses to tumor-derived autophagosomes (DRibbles)
Source: Cell Death Dis. 2016 Aug 4;7(8):e2322–. doi: 10.1038/cddis.2016.206 (PMC5108312; doi:10.1038/cddis.2016.206)
Supplement: Supplementary Figure 1 [file cddis2016206x1.pdf]

Supplementary Figure 1 Preparation of CMV pp65 antigen specific T cell.

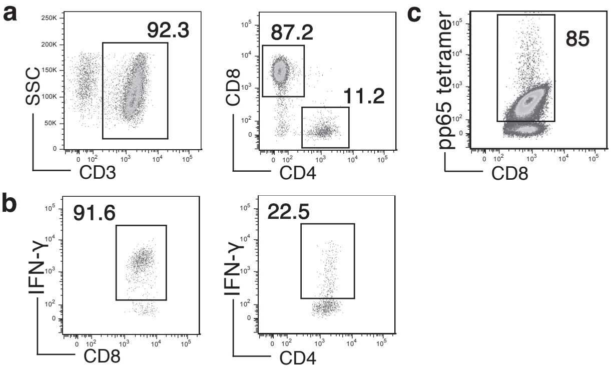

Supplementary Figure 1 Preparation of CMV pp65 antigen specific T cell.
(a) T cells were expanded from PBMCs by priming with pp65 peptides in the presence of human IL-2 (1000 U/ml) for 10–12 days. The cellular phenotype was tested by FACS. (b) Expanded T cells were re-stimulated by pp65 peptides and intracellular IFN-γ was measured by ICS. (c) A high percentage of pp65 antigen-specific CD8+T cells were obtained, as measured by ICS of pp65 tetramer.
