# Supplementary material for: TLR and NLRP3 inflammasome-dependent innate immune responses to tumor-derived autophagosomes (DRibbles)
Source: Cell Death Dis. 2016 Aug 4;7(8):e2322–. doi: 10.1038/cddis.2016.206 (PMC5108312; doi:10.1038/cddis.2016.206)
Supplement: Supplementary Figure 2 [file cddis2016206x2.pdf]

Supplementary Figure 2 Block HSP90 in APC and DRibbles both down regulate antigen specific T cell response.

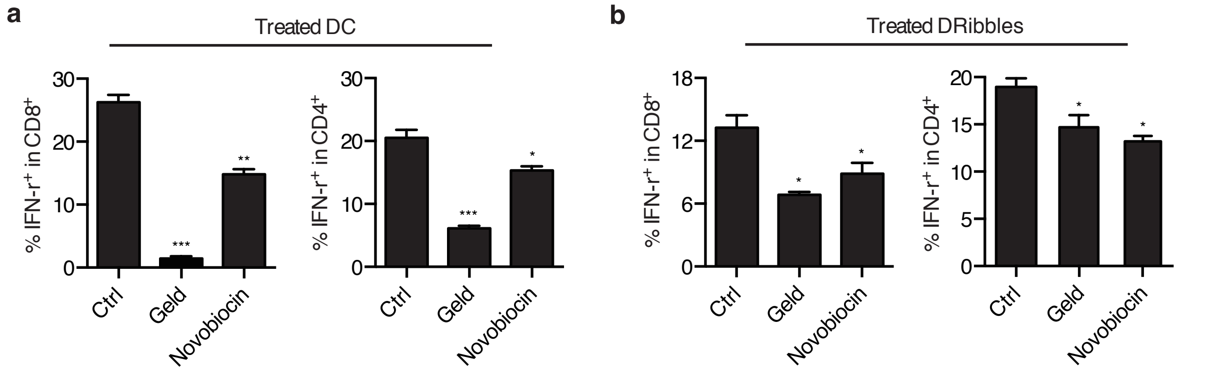

Supplementary Figure 2 Block HSP90 in APC and DRibbles both down regulate antigen specific T cell response.
(a) GM-CSF/IL-4 derived DC was pre-treated with GA and Novobiocin for 30 min, the frequency of IFN-γ+ T cells was detected by ICS after UbiLT3 pp65 DRibbles (30 μg/ml) loaded DC were incubated with expanded T cells. (b) UbiLT3 pp65 DRibbles incubated with GA and Novobiocin for 30 min, the extra inhibitors were washed by PBS. The frequency of IFN-γ+ T cells was detected by ICS after UbiLT3 pp65 DRibbles (30 μg/ml) loaded DC were incubated with expanded T cells. FACS data were analyzed by Flowjo.
